# Demystifying COVID-19 publications: institutions, journals, concepts, and topics

**DOI:** 10.5195/jmla.2021.1141

**Published:** 2021-07-01

**Authors:** Haihua Chen, Jiangping Chen, Huyen Nguyen

**Affiliations:** 1 haihua.chen@unt.edu, PhD Candidate, Department of Information Science, University of North Texas, Denton, TX; 2 jiangping.chen@unt.edu, Professor and Chair, Department of Information Science, University of North Texas, Denton, TX; 3 huyennguyen5@my.unt.edu, Doctoral Student, Department of Information Science, University of North Texas, Denton, TX

**Keywords:** COVID-19 pandemic, CORD-19 dataset, global research roadmap, data analytics, topic modeling

## Abstract

**Objective:**

We analyzed the COVID-19 Open Research Dataset (CORD-19) to understand leading research institutions, collaborations among institutions, major publication venues, key research concepts, and topics covered by pandemic-related research.

**Methods:**

We conducted a descriptive analysis of authors' institutions and relationships, automatic content extraction of key words and phrases from titles and abstracts, and topic modeling and evolution. Data visualization techniques were applied to present the results of the analysis.

**Results:**

We found that leading research institutions on COVID-19 included the Chinese Academy of Sciences, the US National Institutes of Health, and the University of California. Research studies mostly involved collaboration among different institutions at national and international levels. In addition to bioRxiv, major publication venues included journals such as *The BMJ, PLOS One, Journal of Virology*, and *The Lancet*. Key research concepts included the coronavirus, acute respiratory impairments, health care, and social distancing. The ten most popular topics were identified through topic modeling and included human metapneumovirus and livestock, clinical outcomes of severe patients, and risk factors for higher mortality rate.

**Conclusion:**

Data analytics is a powerful approach for quickly processing and understanding large-scale datasets like CORD-19. This approach could help medical librarians, researchers, and the public understand important characteristics of COVID-19 research and could be applied to the analysis of other large datasets.

## INTRODUCTION

COVID-19 is an extremely dangerous disease due to its high infection and death rates and the damage it has caused to the world's economy. The COVID-19 epidemic started as a series of unidentified pneumonia cases reported in Wuhan, China, in late December 2019. On January 30, 2020, the World Health Organization (WHO) officially characterized the disease as a “public health emergency of international concern” or pandemic [1]. The current COVID-19 pandemic is unprecedented. Although some good progress has been made in epidemic preparedness since previous outbreaks over the last decade, there are still clear and significant challenges [2].

To accelerate COVID-19 research and unite the research community in focusing on key knowledge gaps, the WHO in collaboration with the Global Research Collaboration for Infectious Disease Preparedness and Response released *A Coordinated Global Research Roadmap* (herein abbreviated as GRRM) on March 2, 2020 [2]. The GRRM has two goals: (1) to support the diagnosis and treatment of affected patients while facilitating collaboration between different research areas and (2) to accelerate the development of sustainable global research platforms that are prepared for the next future disease pandemic [2]. The GRRM also highlights a group of cross-cutting research priorities, including the human-animal interface, clinical considerations, vaccines, behaviors and education, transmission, therapeutics, health care workers, and ethical considerations [2]. The suggested timeline for implementation of these research priorities extends into early 2021.

In the meantime, the global research community is investing continuous efforts into pandemic-related research areas. Many research studies in transmission, therapeutics, vaccines, and health care have been published since the outbreak. The newly available CORD-19 dataset [3], which is primarily prepared by the Allen Institute for AI, consists of all scholarly papers describing COVID-19 and coronavirus-related research (e.g., SARS, MERS, etc.) from the PubMed Central open-access corpus, WHO's COVID-19 research article corpus, and bioRxiv and medRxiv preprints. On March 16, 2020, the US White House issued a call to action in developing new data and text mining techniques and natural language processing to address scientific questions about COVID-19 using this dataset.

The CORD-19 dataset is a valuable resource for scientists to discover patterns of viral spread, improve diagnostic speed and accuracy, and develop novel effective therapeutic approaches to fight against this novel virus [4]. It is updated daily. On August 5, 2020, the dataset consisted of a metadata file of 207,595 papers in CSV format and a set of 161,297 full-text papers in JSON format. This CORD-19 dataset has been widely used by researchers to investigate medical problems and solutions in the literature. For example, Kaggle held competitions that aimed to extract information on transmission, incubation, environmental stability, and COVID-19 risk factors from this dataset by machine learning algorithms with a human curation overlay [5]. A multilingual Question-Answering & Summary system was built by reading the abstract of all papers in the dataset based on the BERT model, a pretrained language model for word representation, and Google translation [6]. Furthermore, Fister et al. used the association rule text mining and information cartography techniques to extract structured knowledge from the abstracts of papers in the dataset to understand how researchers responded in similar epidemic/pandemic situations throughout history [7].

For information professionals and medical librarians, understanding this dataset is valuable for seeing the big picture of COVID-19 research to better assist users in finding and using relevant information.

The purpose of this study was to understand existing research efforts combating COVID-19 through a large-scale analysis of CORD-19. Specifically, we aimed to answer the following three questions:

1. What are the major characteristics of COVID-19 literature as reflected in the CORD-19 dataset?
2. What are the major research topics or areas of focus investigated by research communities?
3. How have these research topics evolved over time?

To answer these questions, we conducted descriptive analysis and a series of automatic data analysis of metadata records, including key word extraction, Latent Dirichlet Allocation (LDA) topic modeling, and topic evolution.

## METHODS

There are several ways to analyze a collection of text data depending on the purpose of the analysis [8]. Manually analyzing a large dataset like CORD-19, however, is not feasible. Automatic text analysis, which incorporates different levels of natural language processing, has been widely applied. We first reorganized and preprocessed the CORD-19 dataset based on our research purpose and then performed two types of text analyses: descriptive analysis and automatic content analysis. Moreover, different visualization techniques were applied for presenting the results.

### Data preparation

We performed data integration, segmentation, and cleaning to prepare the data for processing.

We extracted affiliation information, which was not available in the metadata file, from the set of JSON-formatted files. This attribute was then integrated as a feature in the metadata file.

To understand how research areas evolved over time, we segmented the whole data collection into seven subsets based on monthly publication dates as described in Appendix A. From the first CORD-19 release (on March 13, 2020), we separated publications before 2020 to form subset 0 and after 2020 to create subset 1. The other subsets were formed by excluding papers published before 2020 in each data release and then filtering out duplicates that were released in previous months. Note that the number of publications before 2020 increased with subsequent data releases, which explains why the sum of papers in all subsets does not match the total number of papers in the CORD-19 dataset released on August 5, 2020.

For data cleaning, as suggested by Almuhaideb and Menai [9], we lowercased and removed nonalphabet and stop words from the abstracts and full texts prior to conducting most data analysis tasks. Also, we performed stemming and lemmatization for topic modeling and key word extraction.

### Descriptive analysis

Descriptive analysis of the dataset focused on determining the research institutions of authors, geographical distribution of institutions, and collaboration among institutions from different countries and regions. Also, we identified the leading publication venues and disciplines.

To build an affiliation collaboration network, we counted all collaborations (no duplicates) if there were two or more different affiliations within the same papers. However, the large dimensions of the network made it challenging to visualize all institutions and collaborations. To maximize the visual effect of the network, we set a threshold of thirty-seven collaborations, meaning that only institutions that collaborated with at least thirty-seven other distinct institutions would be included in the network visualization. We compared two popular tools—Python's NetworkX package [10] and Gephi software [11]—for network visualization and chose Gephi, as it is more suitable for visualizing large-scale graphs. Several algorithms were embedded in Gephi to display the spatialization process, which is more flexible to meet the more complicated requirements of users.

### Automatic content analysis

Multiple methods, such as key word or concept extraction and topic modeling, are applied to discover topics covered in publications. Moreover, topic evolution is performed to investigate how topics change over time.

#### Automatic key word or concept extraction.

We considered key words or concepts to be English words or phrases. Many techniques and tools can be used for automatic key word extraction [12–16]; however, not all of them are reusable. Compared to other methods [12, 14–16], YAKE! achieved the highest performance in terms of collections of different sizes and domains [13]. This strengthened our belief that YAKE! would be the most appropriate key word extraction tool for the CORD-19 dataset. YAKE! defines a set of features (casing, position, frequency, relatedness to context, and dispersion of a specific term) capturing key word characteristics, which are heuristically combined to assign a single score to every key word [13]. One limitation of YAKE! is that it takes much more time to run than other tools. Therefore, we only ran YAKE! on a random sample of 20% of the dataset. From this sample, we extracted the top forty concepts or key words that were text sequences with no more than three words.

#### Topic modeling.

Topic modeling is an unsupervised learning model for generating topics from many documents. This method enables us to rapidly generate a comprehensive overview of the content of documents in our huge dataset. Latent Semantic Analysis (LSA) and Latent Dirichlet Allocation (LDA) are the most popular techniques for topic modeling. Compared to LSA, LDA can improve the mixture models that capture the exchangeability of both words and documents from the former method produced by LSA [17]. Therefore, we decided to apply LDA using the Python Gensim LDA package [18].

One of the most important steps for applying topic modeling is to select an appropriate number of topics contained by the corpus. This is difficult sometimes because choosing too few topics will produce results that are overly broad, while choosing too many will result in overclustering, which means the documents in the corpus are classified into many small and overlapping topics [19]. We applied the coherence measurement, which has been widely used to evaluate the quality of topics generated by topic modeling [20], to determine the number of topics (k) for our dataset. Topic coherence scores a single topic by measuring the degree of semantic similarity between high scoring words in the topic. Low quality topics may be composed of highly unrelated words that cannot fit into another topic, leading a low coherence score. Topic coherence measurements help to distinguish topics that are semantically interpretable from those that are artifacts of statistical inference [21]. The higher the coherence score, the higher the quality of the generated topics [20]. We conducted a preliminary experiment on 100 COVID-related documents, with the number of topics set as 10, 15, 20, and 25. If the selected k is too big (i.e., k>25), topic interpretation becomes problematic. The coherence scores were as follows: 0.5131, 0.5115, 0.5055, and 0.5086, respectively. For the highest coherence score, we finally selected k=10.

#### Topic evolution.

Topic evolution refers to changes in the topics and their popularity over time. Here, we examined key word evolution and dynamic topic modeling (DTM) [22] in the seven time-sliced subsets. DTM is the most appropriate approach for analyzing the time evolution of topics in large document collections. State space models are used to represent the topics, and variational approximations are developed to carry out approximate posterior inference over the latent topics [22]. DTM in the Python Gensim package [23] was implemented to generate topics in different time slices, and the method mentioned in Karpovich et al. [24] was reused to visualize topic changes.

## RESULTS

### Research institutions

Based on the affiliations of authors, we found that 2,082 institutions contributed at least 10 papers related to COVID-19. The top 20 institutions involved in COVID-19 research are presented in Table 1.

**Table 1:** The top 20 institutions participating in COVID-19 research

Rank	Institution	Number of documents/publications
**1**	Chinese Academy of Sciences	1,149
**2**	National Institutes of Health	797
**3**	University of California	722
**4**	Huazhong University of Science and Technology	653
**5**	The University of Hong Kong	544
**6**	Chinese Academy of Agricultural Sciences	544
**7**	University of Oxford	517
**8**	Wuhan University	505
**9**	University of Washington	486
**10**	Zhejiang University	464
**11**	Harvard Medical School	421
**12**	Fudan University	415
**13**	Utrecht University	358
**14**	University of Toronto	353
**15**	Huazhong Agricultural University	341
**16**	National Institute of Infectious Diseases	323
**17**	Sichuan University	307
**18**	Stanford University	302
**19**	The Ohio State University	300
**20**	Imperial College London	297

The Chinese Academy of Sciences, the US National Institutes of Health, and the University of California were the three most active institutions. As one of the largest research organizations in the world, the Chinese Academy of Sciences has many branches or subinstitutions in different fields that can conduct COVID-19 research from multiple aspects. Similarly, the National Institutes of Health plays an important role in medical research. The University of California was the leading academic site in the fight against COVID-19. Our analysis also indicates that among the top 100 institutions involved in COVID-19 research, 40 were from the United States and 27 were from China, which aligns with the fact that the pandemic started in China and has hit hardest in the United States. Moreover, both the United States and China are well-known around the world for their large medical research resources [25]. Among the top 100 leading countries/regions, the United Kingdom made the largest contribution after the United States and China. The analysis also indicates the participation of the rest of the world; however, this is incomparable given that 86% contributed only two or fewer COVID-19 publications. The GRRM suggests that countries all over the world should unite to push COVID-19 research forward.

### Institutional collaboration

**Figure 1:**
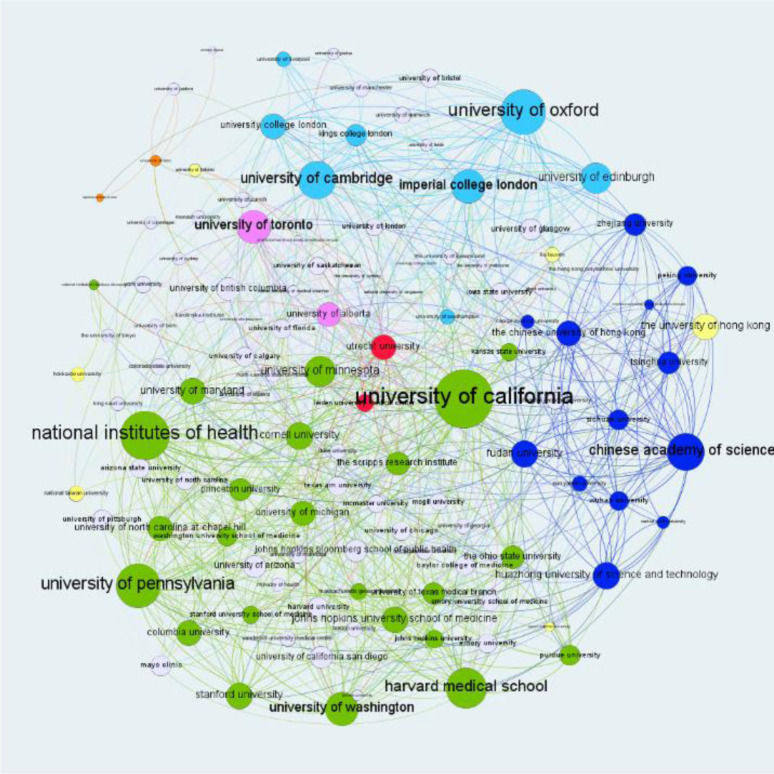
The institution collaboration network. Colors indicate country/region: blue—China, green—United States, light blue—United Kingdom, pink—Canada, yellow—Hong Kong, and red—Italy

We constructed a collaboration network to illustrate collaborations or coauthorship relations among institutions. In Figure 1, nodes represent institutions contributing to COVID-19 research, and lines connecting the nodes represent collaborations between institutions. The size of nodes illustrates the number of collaborations for the institution, and the thickness of lines connecting two nodes indicates how often those two institutions collaborated. Node colors represent the countries/regions of the institutions. Figure 1 shows that the global pandemic motivated collaborations of institutions all over the world. The figure, however, cannot tell the types of collaborations. Further analysis will be needed to understand the types of collaborations and to determine the degree to which the collaborations are domestic or international. We analyzed the Chinese Academy of Sciences as an example and found that even though it had the most collaborations, its proportion of international collaborations was very low (around 8%).

### Publication venues

Our analysis showed that research in the CORD-19 dataset covered about 18,569 publication venues, the majority of which were medical and biology journals. Table 2 lists the top twenty venues. Except for *Nature, Science Report*, and *Science*, which have general scopes covering multiple fields, the remainder belonged to the medical and biology fields, more specifically virology. In addition, by referring to the list of bioinformatics journals at bioinformatics.org [26], we found that thirty-one out of seventy-three journals on this list published articles on COVID-19 research. However, the information science community could make a greater contribution to pandemic research efforts from the perspective of information literacy, sentiment analysis, information recommendation, information-seeking behavior, and archiving of COVID-19 digital resources using various methods including user study, social media analysis, content analysis, or big data analysis. This is aligned with the GRRM's expected role of social science in the outbreak response [2].

**Table 2:** The top 20 publication venues for COVID-19-related research

Rank	Journal/publication venue	Frequency
**1**	bioRxiv	2,129
**2**	The BMJ	2,104
**3**	PLOS One	1,934
**4**	Journal of Virology	1,662
**5**	The Lancet	1,274
**6**	Nature	1,045
**7**	Virology	943
**8**	Surgical Endoscopy	923
**9**	Emerging Infectious Diseases	912
**10**	Journal of Medical Virology	899
**11**	Scientific Reports	830
**12**	Viruses	765
**13**	JAMA	721
**14**	International Journal of Infectious Diseases	701
**15**	Archives of Virology	682
**16**	Science	680
**17**	Critical Care	679
**18**	Journal of Clinical Virology	632
**19**	Vaccine	611
**20**	The Journal of General Virology	595

### Top key words/concepts

To understand the most important research concepts in the publications, we extracted a list of key words (i.e., words or phrases that represent concepts) using YAKE! [13] from the whole dataset. Table 3 presents the top forty important key words that appeared in the dataset, together with their respective scores/weights. The scores in the third column were calculated by YAKE! based on several features of the term, such as term casing, term frequency normalization, number of co-occurring terms, position of sentences, and number of different sentences in which the term occurs [13]. The lower the score, the more important the term.

**Table 3:** The top 40 key words extracted from the dataset

Importance rank	Key word/concept	Score
**1**	respiratory syndrome coronavirus	0.0000000136
**2**	severe acute respiratory	0.0000000226
**3**	acute respiratory syndrome	0.0000000233
**4**	world health organization	0.0000000238
**5**	middle east respiratory	0.0000000581
**6**	east respiratory syndrome	0.0000000711
**7**	patient coronavirus disease	0.0000001580
**8**	coronavirus disease	0.0000001720
**9**	public health emergency	0.0000003310
**10**	health organization march	0.0000003460
**11**	hospital wuhan university	0.0000003630
**12**	health care system	0.0000003770
**13**	hubei province china	0.0000003930
**14**	pandemic world health	0.0000003960
**15**	wuhan city china	0.0000004040
**16**	acute respiratory	0.0000004160
**17**	respiratory syndrome	0.0000004340
**18**	background coronavirus disease	0.0000004440
**19**	syndrome coronavirus	0.0000004490
**20**	public health	0.0000004510
**21**	outbreak coronavirus disease	0.0000004800
**22**	city wuhan china	0.0000005150
**23**	wuhan china december	0.0000005460
**24**	severe coronavirus disease	0.0000006390
**25**	case coronavirus disease	0.0000006690
**26**	global public health	0.0000006850
**27**	world health	0.0000006860
**28**	pandemic coronavirus disease	0.0000006950
**29**	health care	0.0000007380
**30**	wuhan province china	0.0000007390
**31**	electronic supplementary material	0.0000007410
**32**	severe acute	0.0000007420
**33**	respiratory distress syndrome	0.0000007480
**34**	china world health	0.0000007630
**35**	health organization	0.0000007870
**36**	centers disease control	0.0000008010
**37**	severe respiratory disease	0.0000008290
**38**	wuhan university january	0.0000008310
**39**	result coronavirus disease	0.0000008330
**40**	acute respiratory distress	0.0000008430

Table 3 indicates that respiratory syndrome coronavirus, severe acute respiratory, acute respiratory syndrome, world health organization, and middle east respiratory were the five most important key words from the corpus. However, the key words in Table 3 do not match the urgent COVID-19 research topics released by WHO [2], as concepts such as vaccines, behaviors and education, transmission, therapeutics, health care workers, and ethical considerations do not appear to be covered. This may be due to two reasons: (1) these topics are not or hardly covered by the literature, and (2) terms used by authors may not be the same as those used by WHO.

### Topics explored by researchers

The topics/research areas of the publications may not be fully represented by the extracted key words due to their low frequency counts in the dataset. We therefore decided to complement the key word extraction approach with topic modeling. Table 4 lists the most frequently identified terms for each of ten topics as well as a topic summary phrase.

**Table 4:** Global topics inferred from the entire CORD-19 dataset

Topic ID	Word list	Annotated topic
**Topic 1**	calf, food, temperate, product, animal, bovine, HMPV, herd, farm, cattle, feed, energy	Human metapneumovirus (HMPV) and livestock
**Topic 2**	patient, hospital, admission, outcome, clinic, day, 2020, care, severe, mortal, admit, intense	Clinical outcomes of severe patients
**Topic 3**	risk, association, study, factor, higher, increase, compare, result, rate, mortal, year, level	Risk factors of higher mortality rate
**Topic 4**	test, detect, sample, assay, posit, use, result, method, sensitive, diagnostic, RT-PCR, specific	COVID-19 RT-PCR test
**Topic 5**	sequence, genome, gene, region, mutation, protein, nucleotide, recombine, acid, amino, analysis, site	Gene mutation analysis
**Topic 6**	bacterial, bacteria, dog, antibiotic, pathogen, antimicrobial, resistance, canine, coli, microbiology, parasite, disinfect	Antimicrobial-resistant bacteria in dog
**Topic 7**	disease, treatment, drug, review, infect, effect, clinic, develop, potential, therapy, therapeutic, current	Potential therapeutic drugs for COVID-19
**Topic 8**	ACE2, diabetic, kidney, cardiovascular, enzyme, receptor, camel, hypertension, angiotensin, renal, angiotensin-converting, inhibitor	Angiotensin-Converting Enzyme 2 (ACE2)
**Topic 9**	lung, ventilator, injury, pulmonary, pressure, airway, oxygen, mechanical, ARDS, respiratory, cardiac, failure	Acute Respiratory Distress Syndrome (ARDS)
**Topic 10**	transmission, virus, exposure, aerosol, particle, contaminated, infect, environment, water, environment, surface, concentration	Transmission of COVID-19 virus

The topic modeling results indicate that the research community focused on learning about the disease at three levels: (1) characteristics of the disease, including gene mutation, transmission, and human-animal interface; (2) how it affects the human body, such as risk factors, susceptible populations, symptoms, and effects on human mechanisms; and (3) therapeutics and vaccines for the disease. Still, topics such as behaviors and education among the public and ethical issues in pandemic response have not been explored much in the literature, although GRRM recommends investigation of these topics.

### Topic evolution

As listed in Appendix A, we divided the whole collection into seven subsets. Except for the first subset before 2020, the rest of the six slices are roughly in line with the timeline specified in GRRM before August 2020.

Using NEViewer [27] and DTM [22], we conducted key word and topic evolution analysis. When we compared results from NEViewer and DTM, we found that the visualization generated by NEViewer was too confusing due to the overlapping of key words. Therefore, we only present the results from DTM.

**Figure 2:**
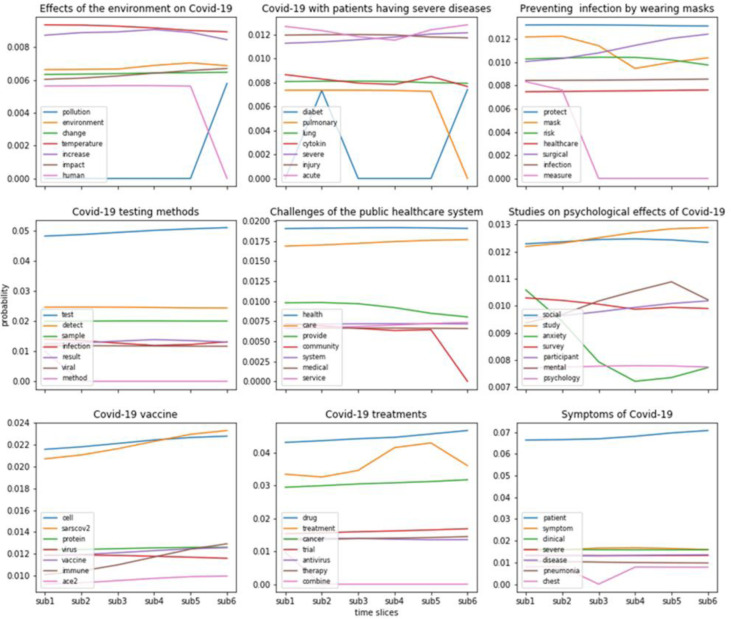
Nine of the most typical topics evolving over six time slices

Figure 2 presents nine of the most typical topics evolving over six time slices from DTM. It consists of nine diagrams, one for each topic. The topics for each diagram are represented by the most frequent meaningful terms or key words. In the diagrams, the legend presents the key words for that topic. The x-axis represents the six time slices, and the y-axis represents the probability of each term. Note that the probability scales in y-axis differ across diagrams. The lines present the probability of individual words or concepts varying over the six time-sliced subsets.

Figure 2 showed that some concepts or key words, such as symptom, severe acute, protection, COVID-19 testing, public health, vaccine, and treatment, have high occurrence over time. It also indicates that as people learned more about the disease, attention to some topics decreased, such as pulmonary, community infection, and social anxiety. Therefore, as time goes on, the research community may have achieved a better understanding of these topics.

## DISCUSSION

By performing affiliation and publication venue analysis, we identified some basic characteristics of the whole CORD-19 dataset [28–29] to answer the first research question regarding the major characteristics of COVID-19 literature. Our analysis indicates that researchers in many institutions all over the world have participated in the war against the pandemic. Prominent research institutions evidently contributed in a major way to the work. Also, there appears to be a lack of international collaborations in the COVID-19 research network. More international collaborations may have the potential to speed up the progress of COVID-19 research. Also, although academic journals are major venues for researchers to share knowledge discovery and results, our results underscore that preprint servers and platforms such as bioRxiv allow fast distribution of research results and have become widely accepted by researchers.

The second research question regarding the major research topics or areas of focus was addressed by applying several methods to determine research areas and extract topics and key words from the COVID-19 literature. We found that while the major research areas were biology and medicine, COVID-19-related research was also published in social science journals. However, social science–related issues were not among the ten most prominent global topics of COVID-19. Topics extracted from the entire dataset mainly covered biological characteristics of the SARS-CoV-2 virus, such as genetics, testing, therapeutics, transmission, and symptoms. By contrast, visualization of the six time-sliced segments presents more multi-faceted topics of the pandemic than topic modeling. In addition to the biological features of the disease, topic evolution analysis discovered topics such as psychological impacts, environmental outcomes, and the effects of wearing masks.

For the third research question, we found that research focuses changed substantially over time slices, probably based on the current situation and progress of COVID-19 research. Some prioritized research topics recommended by the GRRM were highly concerted and investigated by the research community, such as treatment, vaccine, infection prevention, and diagnostics. However, a few important aspects of the COVID-19 pandemic have not received enough attention: virus origin, health care worker protection, human-animal interface, and ethical issues. Our findings could benefit the research community by helping it adjust future research focuses and thereby fight the pandemic more effectively.

This study is innovative as we combined multiple methods to answer each research question to account for the strengths and weaknesses of each of the automatic methods applied. Python programming language was used to run most of the analytic tasks in combination with other tools, and multiple visualization tools were used to save time and maximize the visual effect of the results. For example, Gephi and NEViewer were used to visualize our outputs. Tools with a good user interface, available color palettes, and integrated algorithms were hereby deemed to have higher usability and proved to be timesaving. However, the selection of tools depends on the analytic tasks; for example, though NEViewer software allows users the capability to interact with data, color, and other attributes, the graph generated to visualize topic and key word evolutions was less interpretable. Our analytic experience with these tools can be applied for analyzing comparable datasets or solving similar analytic problems.

We believe health sciences librarians and information professionals could benefit in multiple ways from this study. First, we identified and applied effective data analytic tools, such as YAKE!, LDA, DTM, and Gephi. These tools can be learned and used by health sciences information professionals to analyze large text collection automatically. Second, health sciences information professionals can adjust their research directions by focusing on urgent but not well-studied topics based on our findings. Moreover, top institutions and publication venues for COVID-19-related research discovered in this study may help health sciences information professionals identify potential collaborators and target publication venues for their services and collection development.

This study has its limitations. As the CORD-19 dataset is continuously updated every day, the results may not be accurate in the future. However, our methods can be replicated to investigate future growth of datasets. Furthermore, the dataset is very large, so for some methods, such as YAKE! key word extraction, we were not able to run the program on the entire dataset but only a random sample of the dataset. Although the sample was randomly selected, the results might not represent the true findings for the entire dataset.

In conclusion, this study provides a comprehensive understanding of the continuous efforts of the research community to defeat the global pandemic. It shows that the current COVID-19 research direction partly aligns with the research roadmap released by the WHO [2]; some research topics have not been fully studied, such as health care worker protection, human-animal interface, and ethical issues. Therefore, the COVID-19 research community may want to adjust their research direction to respond properly to the pandemic. Our study may benefit health information professionals who are dealing with an overload of information about the pandemic and the disease and could be used as a resource in providing information services. Furthermore, our method can be reused to analyze other large datasets.

In the future, we will continue to track the progress in COVID-19 research. For example, we are working on developing a knowledge graph of diseases based on the same dataset, which will provide an effective approach to organize knowledge included in the dataset for better information access and use.

## Data Availability

Programs and data files are available for research use in GitHub (https://github.com/HuyenNguyenHelen/JMLA_CORD19-DS).

## SUPPLEMENTAL FILES

**Appendix A:** The seven subsets divided into time series
