# Supplementary material for: Demystifying COVID-19 publications: institutions, journals, concepts, and topics
Source: J Med Libr Assoc. 2021 Jul 1;109(3):395–405. doi: 10.5195/jmla.2021.1141 (PMC8485960; doi:10.5195/jmla.2021.1141)
Supplement: Supplementary file 1 — Appendix A: The seven subsets divided into time series [file jmla-109-3-395-s01.docx]

**APPENDIX A.** The seven subsets divided into time series

| **Subset** | **Time span** | **Total documents** | **Total full-text documents** |
| --- | --- | --- | --- |
| Sub-0 | Before 2020 | 27,453 | 12,381 |
| Sub-1 | Jan 1–Mar 13 | 2,047 | 838 |
| Sub-2 | Mar 14–Apr 3 | 2,603 | 1,836 |
| Sub-3 | Apr 4–May 12 | 9,395 | 7,955 |
| Sub-4 | May 13–Jun 5 | 35,647 | 12,401 |
| Sub-5 | Jun 6–Jul 5 | 44,354 | 10,764 |
| Sub-6 | Jul 6–Aug 5 | 24,913 | 9,507 |
| **Total** | | **146,412** | **55,682** |
